# Spatial relationship between mitral valve and ventricular septum assessed by resting echocardiography to diagnose left ventricular outflow tract obstruction in hypertrophic cardiomyopathy

**DOI:** 10.1093/ehjci/jead036

**Published:** 2023-02-26

**Authors:** Nicolas Verheyen, Angelika Batzner, David Zach, Andreas Zirlik, Brenda Gerull, Stefan Frantz, Christoph Maack, Stefan Störk, Hubert Seggewiss, Caroline Morbach

**Affiliations:** Division of Cardiology, University Heart Center, Department of Internal Medicine, Medical University of Graz, Auenbruggerplatz 15, 8036 Graz, Austria; Comprehensive Heart Failure Center, University Hospital Würzburg, Am Schwarzenberg 15, 97078 Würzburg, Germany; Department of Internal Medicine I, University Hospital Würzburg, Oberdürrbacher Straße 6, Haus A15, 97080 Würzburg, Germany; Comprehensive Heart Failure Center, University Hospital Würzburg, Am Schwarzenberg 15, 97078 Würzburg, Germany; Department of Internal Medicine I, University Hospital Würzburg, Oberdürrbacher Straße 6, Haus A15, 97080 Würzburg, Germany; Division of Cardiology, University Heart Center, Department of Internal Medicine, Medical University of Graz, Auenbruggerplatz 15, 8036 Graz, Austria; Division of Cardiology, University Heart Center, Department of Internal Medicine, Medical University of Graz, Auenbruggerplatz 15, 8036 Graz, Austria; Comprehensive Heart Failure Center, University Hospital Würzburg, Am Schwarzenberg 15, 97078 Würzburg, Germany; Department of Internal Medicine I, University Hospital Würzburg, Oberdürrbacher Straße 6, Haus A15, 97080 Würzburg, Germany; Comprehensive Heart Failure Center, University Hospital Würzburg, Am Schwarzenberg 15, 97078 Würzburg, Germany; Department of Internal Medicine I, University Hospital Würzburg, Oberdürrbacher Straße 6, Haus A15, 97080 Würzburg, Germany; Comprehensive Heart Failure Center, University Hospital Würzburg, Am Schwarzenberg 15, 97078 Würzburg, Germany; Department of Internal Medicine I, University Hospital Würzburg, Oberdürrbacher Straße 6, Haus A15, 97080 Würzburg, Germany; Comprehensive Heart Failure Center, University Hospital Würzburg, Am Schwarzenberg 15, 97078 Würzburg, Germany; Department of Internal Medicine I, University Hospital Würzburg, Oberdürrbacher Straße 6, Haus A15, 97080 Würzburg, Germany; Comprehensive Heart Failure Center, University Hospital Würzburg, Am Schwarzenberg 15, 97078 Würzburg, Germany; Department of Internal Medicine I, University Hospital Würzburg, Oberdürrbacher Straße 6, Haus A15, 97080 Würzburg, Germany; Comprehensive Heart Failure Center, University Hospital Würzburg, Am Schwarzenberg 15, 97078 Würzburg, Germany; Department of Internal Medicine I, University Hospital Würzburg, Oberdürrbacher Straße 6, Haus A15, 97080 Würzburg, Germany

**Keywords:** hypertrophic obstructive cardiomyopathy, transthoracic echocardiography, residual mitral leaflet, ventricular outflow tract obstruction, provocable obstruction, tip-to-septum distance

## Abstract

**Aims:**

Echocardiographic diagnosis of left ventricular outflow tract obstruction (LVOTO) in hypertrophic cardiomyopathy (HCM) often requires extensive provocative manoeuvers. We investigated, whether echocardiography-derived parameters obtained at rest can aid to determine the presence of LVOTO in persons with HCM.

**Methods and results:**

Consecutive patients with HCM admitted to a referral centre underwent standardized transthoracic echocardiographic examination including provocative manoeuvers. Under resting conditions, the length of mitral leaflets and distances between mitral valve coordinates and ventricular walls were blindly measured in parasternal long axis (PLAX) and apical three-chamber (3ch) views, both at early and late systole. Among 142 patients (mean age 59 ± 13 years, 42% women), 68 (42%) had resting or provocable LVOTO with maximal LVOT gradients ≥30 mmHg. Late-systolic distance between mitral leaflet tip and ventricular septum (TIS) was measurable in 137 participants (96%) in 3ch view and independently associated with LVOTO in multivariable logistic regression analysis. The area under the ROC curve of TIS for the identification of LVOTO was 0.91 [95% confidence interval (CI) 0.87–0.96]. TIS ≤ 14 mm yielded 97% sensitivity and 57% specificity regarding LVOTO. TIS >14 mm ruled out LVOTO with a negative predictive value of 95%. TIS ≤9 mm ruled in LVOTO with a positive predictive value of 92% (sensitivity 73%, specificity 95%). Among 43 patients with TIS between 10 and 14 mm, 35% had LVOTO.

**Conclusion:**

In our study, the novel echocardiographic parameter TIS showed high negative and positive predictive values for LVOTO in HCM. These exploratory results await confirmation in larger collectives and prospective investigations.

## Graphical Abstract

**Graphical Abstract:**
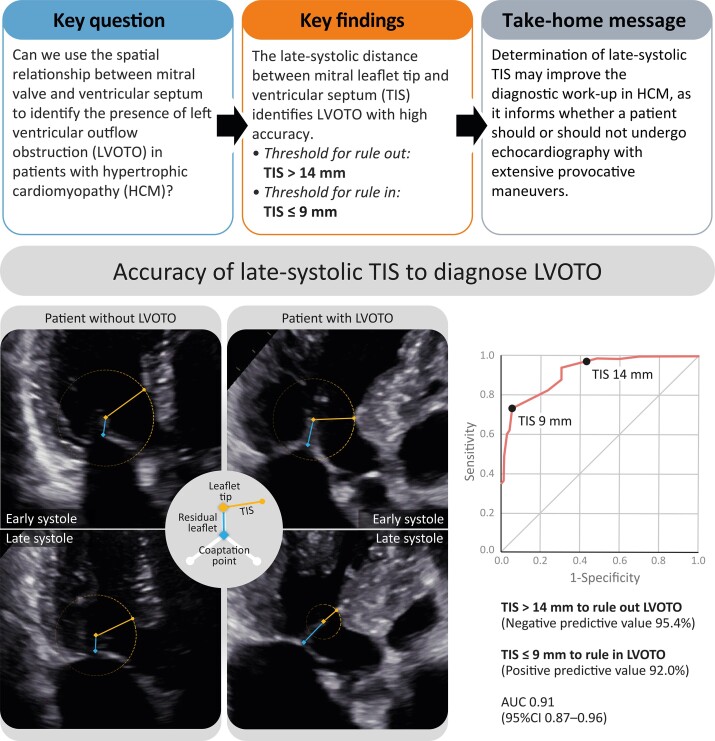
Diagnostic accuracy of late-systolic mitral leaflet tip-to-septum distance to identify left ventricular outflow tract obstruction in hypertrophic cardiomyopathy. Left: echocardiographic images of two representative patients with HCM (left: without LVOTO; right: with LVOTO), with spatial relationship parameters corresponding to mean values of the studied patient sample. Blue diamond, coaptation point; yellow diamond, mitral leaflet tip; blue line, residual leaflet; yellow line, TIS. Circular compass line indicates that TIS is defined as the shortest distance between leaflet tip and ventricular septum. AUC, area under the ROC curve; CI, confidence interval; LVOTO, left ventricular outflow tract obstruction; NPV, negative predictive value; PPV, positive predictive value; Sens, sensitivity; Spec, specificity; TIS, distance between mitral leaflet tip and ventricular septum. Right: Receiver operating characteristic curve analysis depicting the diagnostic accuracy of late-systolic TIS measured at rest in apical three chamber view to identify the presence of LVOTO in patients with HCM (inverse association). Thresholds for optimal sensitivity (TIS >14 mm to rule out LVOTO) and specificity (TIS ≤9 mm to rule in LVOTO) are indicated.

**See the editorial comment for this article ‘A new index for the diagnosis of outflow obstruction in hypertrophic cardiomyopathy: is it accurate? and if so, is it useful?’, by P. Spirito and L. Boni, https://doi.org/10.1093/ehjci/jead055.**

## Introduction

Hypertrophic cardiomyopathy (HCM) is a hereditary cardiomyopathy with an estimated prevalence of up to 0.6% in the general population.^1^ Left ventricular outflow tract obstruction (LVOTO) is present in up to 70% of patients with HCM and increases the risk of heart failure and death.^2,3^ Once LVOTO is diagnosed, pharmacological and invasive therapies are available for its treatment.^4–7^ Also after septal reduction therapy (SRT), residual LVOTO can be present.^8^ The severity of LVOTO is affected by pre- and afterload conditions, ventricular morphology, alterations of the mitral valve apparatus, and ventricular contractility, and about 50% of LVOTO patients exhibit a significant obstruction only during provocative manoeuvers.^3^ Therefore, an extensive sequence of examination steps including standardized provocative manoeuvers is recommended during echocardiography in patients with HCM and a left ventricular outflow tract (LVOT) gradient <30 mmHg to reliably exclude or identify LVOTO.^9^ Given that awareness of the dynamic component of LVOTO and experience with provocative manoeuvers are often limited in clinical practice, there is substantial underdiagnosis of LVOTO with an unacceptably high proportion of patients being withheld from optimal treatment.^10^ An easily applicable echocardiographic parameter to discriminate between obstructive and non-obstructive HCM is therefore desirable.

Systolic anterior motion (SAM) of the post-coaptational portion of the mitral valve is considered the predominant mechanism leading to LVOTO. However, SAM as semi-quantitative echocardiography-derived parameter is of limited use, due to its high inter-observer variability and poor diagnostic accuracy for identification of LVOTO, particularly regarding provocable LVOTO.^3,11^ Previous studies suggested that both elongation of the post-coaptational portion of the mitral leaflet and the spatial relationship between mitral valve and ventricular walls may determine the presence of LVOTO.^11–13^

We therefore aimed (i) to establish echocardiography-derived parameters reflecting the interaction between the mitral leaflet and the ventricular walls during systole and (ii) to identify the echocardiographic markers with the best diagnostic properties for exclusion and detection of LVOTO in a sample of consecutive patients with HCM including patients with and without previous SRT.

## Methods

This is a cross-sectional analysis of the HyperCard Registry. This registry is a single-centre prospective cohort study conducted at the Comprehensive Heart Failure Centre (CHFC), Würzburg, a referral centre for HCM. The registry includes consecutive patients with diagnosed or suspected HCM, with and without previous SRT. It has been approved by the local Ethics Committee (EC-No. 2020-110 20200526E, first patient in July 2020) and adheres to the principles of Good Clinical Practice and the Declaration of Helsinki. Between October 2020 and September 2021, 183 patients consented for participation in the HyperCard Registry. For the present report, we analysed 147 patients fulfilling diagnostic criteria of HCM with complete digitally stored standardized transthoracic echocardiograms.^9,14^ Patients with previous mitral valve surgery (*n* = 2) and concomitant presence of subaortic membrane (*n* = 3) were excluded. Laboratory analysis, physical examination, medical history, vital parameters, and 12-lead electrocardiogram were assessed at the day of echocardiographic examination.

### Echocardiographic analysis

Transthoracic echocardiography was performed at the CHFC by trained and internally certified sonographers using a Vivid E95 machine and a 4Vc-D probe (GE Vingmed, Horten, Norway).^15^ Echocardiography followed a standardized protocol with acquisition of cine loops over three cardiac cycles in parasternal, apical, and subcostal views using ECG gating.^16^ Still images and loops with a mandated frame rate of at least 55 frames per second were stored digitally. LVOT gradient was measured in apical three- (3ch) and five-chamber (5ch) view at rest and during the strain phase of Valsalva manoeuver in every patient. Continuous-wave Doppler cursor was positioned parallel to the LVOT long axis, and LVOT flow velocity was measured under simultaneous B-mode visualization to facilitate differentiation from mid-cavitary gradients. As recommended, further provocative manoeuvers in addition to Valsalva manoeuver (squat-to-stand, passive leg raising, walking) were conducted in any patient, in whom provocable LVOTO was clinically suspected, e.g. in case of systolic murmur after exercise manoeuver, or if typical symptoms were reported, such as worsening of symptoms after heavy meals or alcohol intake.^9^ Basic echocardiographic parameters were measured offline from digitally stored images and loops by an experienced sonographer of the Academic Core Lab Ultrasound-based Cardiovascular Imaging at the CHFC. The Simpson’s biplane method was used to compute left ventricular ejection fraction (LVEF) and left ventricular stroke volume. Left atrial volume index was determined using the method of discs, and the Dubois formula was applied to calculate body surface area (BSA). Peak early velocities of transmitral filling (E) and lateral mitral annulus (e’ lateral) were measured using Doppler imaging, and their ratio was used to calculate E/e’ lateral.^17,18^

#### HCM-specific parameters

Resting LVOTO was defined as maximal peak LVOT gradient ≥30 mmHg at rest. Provocable LVOTO was defined as a resting gradient <30 mmHg and peak LVOT gradient ≥30 mmHg under provocative manoeuvers.^9,14^ Maximal end-diastolic thickness of the ventricular septum was measured in parasternal long axis. HCM phenotype was categorized into reverse curve, sigmoid, apical, and neutral hypertrophy pattern as defined by Binder and colleagues.^19^ SAM was assessed at rest and was defined as partial or total motion of one of the mitral valve leaflets towards the ventricular septum during systole. SAM was categorized into Grade 0 (no valve motion towards the septum), Grade I (incomplete: valve motion towards the septum, but no septal contact), Grade II (complete: valve motion towards the septum, with contact in late systole), and Grade III (complete: valve motion towards the septum, with contact in early systole). SAM was classified as complete if contact between the mitral valve and the septum was documented in at least one view. We did not differentiate, whether SAM originated from anterior mitral leaflet (AML) or posterior mitral leaflet (PML). Apical papillary muscle displacement was defined as present if the base of the respective papillary muscle originated from the apical third of the left ventricle.^20^

#### Spatial relationship parameters

**Figure 1:**
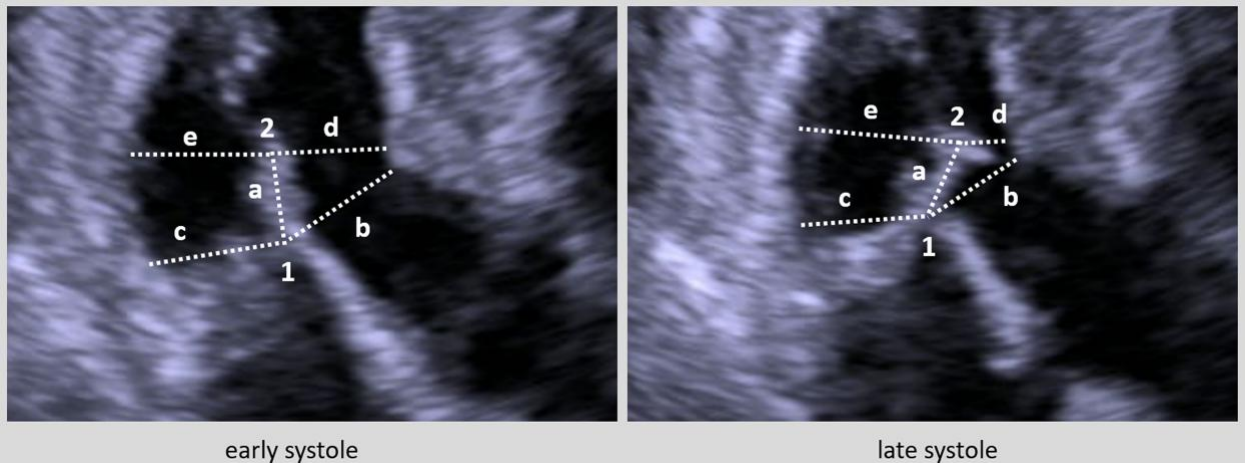
Echocardiographic parameters reflecting the spatial relationship between mitral valve and ventricular walls. (1) Mitral valve coaptation point; (2) mitral leaflet tip; (a) residual leaflet length; (b) closest distance between coaptation point and ventricular septum; (c) closest distance between coaptation point and posterior wall; (d) closest distance between mitral leaflet tip and ventricular septum (TIS); (e) closest distance between mitral leaflet tip and posterior wall.

Parameters were measured off-line from digitally stored images and loops using EchoPAC (GE Healthcare, Chicago, USA), by a cardiologist certified in transthoracic echocardiography by the European Association of Cardiovascular Imaging (NV). Parameters reflecting spatial relationship between mitral valve and ventricular walls were acquired at rest (without provocative manoeuvers) both in 3ch view and in parasternal long axis (PLAX), both at early and late systole. Frames capturing first mitral leaflet coaptation and closest distance between the mitral leaflet tip and the ventricular septum were considered for early-systolic and late-systolic measurements, respectively. Lengths of AML and PML were measured at end diastole. The residual leaflet of the mitral valve (i.e. the post-coaptational portion) was defined as the portion of the mitral valve extending distant the mitral valve coaptation point (*Figure 1*). Distance measures were performed originating from the coaptation point (coaptation point—ventricular septum, coaptation point—posterior wall) and from the mitral leaflet tip (leaflet tip—ventricular septum [TIS], leaflet tip—posterior wall), respectively.

### Data analysis

Patient characteristics were described in the whole sample and stratified by presence of LVOTO. Normal distribution of variables was checked by Q–Q_plots, concordance between mean and median, and skewness. Categorical variables were expressed as count with percentage. Continuous measures were described as mean ± standard deviation or median with quartiles, as appropriate. For group comparisons between presence and absence of LVOTO, we used Mann–Whitney *U* test, chi-square test, and linear-by-linear association, as appropriate. Associations between spatial relationship parameters and presence of LVOTO were assessed using multivariable logistic regression, with adjustment for parameters significantly associated with LVOTO in univariable analysis. Receiver operating characteristic (ROC) curve analyses were applied. Diagnostic accuracy of continuous spatial relationship parameters was expressed as area under the ROC curve (AUC) with 95% confidence interval (CI). Reference thresholds based on optimized sensitivity and specificity were derived from AUC, and diagnostic accuracies of these cut-offs were expressed as diagnostic effectiveness (number of correctly positive and correctly negative subjects divided by the total number). Diagnostic accuracy analyses were repeated in subgroups (no previous SRT, provocable LVOTO, resting LVOTO, women, and men) and with adjustment for BSA and stroke volume. Intra- and inter-observer variabilities were assessed in a random sample of 20 patients calculating intra-class correlation coefficients with 95% CI, and Cohen’s κ for categorical variables. SPSS Statistics Version 27 (IBM, New York, United States) was used for statistical analyses. The significance level was set at 5%.

## Results

### Characteristics of the study sample

We identified 142 consecutive patients with HCM eligible for the present analysis (see Supplementary data online, *Table S1*). Mean age was 59 ± 13 years (range 23 to 84 years), and 59 (42%) were women. Of the total sample, 74 patients (52%) had no LVOTO, and 68 (48%) had LVOTO (*n* = 38 with provocable LVOTO, *n* = 30 with resting LVOTO). SRT had been performed in 59 patients (42%) and 121 patients (85%) were on a beta-blocker therapy.

### Echocardiographic analyses

Late-systolic spatial relationship parameters were measurable in 3ch view in 137 patients (96%) and in PLAX in 115 patients (81%). In all cases with missing parameters, this was due to inadequate spatial resolution. Due to higher feasibility, we report 3ch view-derived parameters, only.

LVOTO was associated with higher LVEF, higher septal thickness, the degree of SAM, and apical displacement of both anterolateral and posteromedial papillary muscle. Basic echocardiographic parameters stratified by LVOTO are shown in *Table 1*. In multivariable logistic regression analyses, LVOTO was associated with longer residual mitral leaflets (for both early- and late-systolic measurements), shorter TIS (for both early- and late-systolic measurements), and longer distances between mitral leaflet tip and the posterior wall (only for late-systolic measurement), as shown in *Table 2*. Analyses were adjusted for sex, NYHA functional class, atrial fibrillation, device therapy, LVEF, degree of SAM, maximal septal thickness, apical displacement of anterolateral papillary muscle, and apical displacement of posteromedial papillary muscle.

**Table 1:** Basic echocardiographic parameters of the total sample and stratified by left ventricular outflow tract obstruction

	All subjects *N* = 142	No LVOTO *n* = 74	LVOTO *n* = 68	*P*-value
LVEF, %	68 (8)	67 (8)	70 (8)	0.025
LAVI, mL/m²	44 (34–54)	43 (33–55)	45 (35–53)	0.532
E/e’ lateral#	10.6 (8.4–14.3)	11.0 (8.5–15.1)	13.4 (9.1–16.6)	0.195
IVSed_max_, PLAX, mm	21 (5)	19 (4)	22 (4)	<0.001
SAM, *n* (%)				<0.001
ȃ0	57 (40)	51 (69)	6 (9)	
ȃI	64 (45)	23 (31)	41 (60)	
ȃII	21 (15)	0 (0)	21 (31)	
ȃIII	0 (0)	0 (0)	0 (0)	
Peak LVOT gradient, mmHg				
ȃAt rest	12 (7–21)	8 (6–10)	23 (15–45)	<0.001
ȃValsalva	18 (9–57)	10 (6–16)	59 (38–107)	<0.001
ȃMaximal gradient	28 (10–80)	11 (7–17)	84 (47–125)	<0.001
HCM phenotype, *n* (%)				0.772
ȃReverse curve	114 (80)	56 (76)	58 (85)	
ȃSigmoid	18 (13)	11 (15)	7 (10)	
ȃApical	6 (4)	6 (8)	0 (0)	
ȃNeutral	4 (3)	1 (1)	3 (5)	
Apical papillary muscle displacement, *n* (%)				
ȃAnterolateral	12 (9)	3 (4)	9 (13)	0.049
ȃPosteromedial	47 (33)	31 (42)	16 (24)	0.023

# more than mild mitral annular calcification excluded (*n* = 20).

Data are frequency (%), mean (standard deviation), or median (quartiles).

*P*-value for group comparison between patients with vs. without LVOTO.

HCM, hypertrophic cardiomyopathy; IVSed_max_, maximal end-diastolic thickness of interventricular septum; LAVI, left atrial volume index; LVEF, left ventricular ejection fraction, LVOTO, left ventricular outflow tract obstruction; PLAX, parasternal long axis; PW, posterior wall; SAM, systolic anterior motion of the mitral valve.

**Table 2:** Echocardiography-derived mitral valve parameters in the total sample and association with left ventricular outflow tract obstruction in univariable and multivariable logistic regression analyses

		All subjects *N* = 142	Univariable group comparison			Multivariable logistic regression analysis	
			No LVOTO *n* = 74	LVOTO *n* = 68	*P*-value	OR (95% confidence interval)	*P*-value
**Lengths, mm**							
Complete AML		29 (4)	28 (4)	30 (4)	0.008	1.090 (0.933–1.273)	0.277
Complete PML		21 (4)	20 (3)	23 (4)	<0.001	1.122 (0.918–1.371)	0.262
Residual mitral leaflet	early-systolic	9 (7–12)	7 (6–9)	11 (8–14)	<0.001	1.388 (1.076–1.791)	0.012
	late-systolic	10 (7–13)	8 (6–11)	13 (10–15)	<0.001	1.266 (1.030–1.557)	0.025
**Distances, mm**							
Coaptation point—ventricular septum	early-systolic	22 (19–25)	24 (21–26)	21 (18–24)	0.006	0.896 (0.766–1.048)	0.169
	late-systolic	18 (16–22)	20 (17–22)	17 (14–19)	<0.001	0.973 (0.841–1.125)	0.708
Coaptation point—posterior wall	early-systolic	18 (15–20)	17 (15–20)	19 (16–21)	0.750	0.953 (0.827–1.099)	0.510
	late-systolic	12 (10–15)	12 (10–14)	13 (11–16)	0.184	0.954 (0.821–1.109)	0.542
Mitral leaflet tip—ventricular septum (TIS)	early-systolic	19 (16–23)	22 (18–25)	18 (15–21)	<0.001	0.867 (0.756–0.993)	0.040
	late-systolic	11 (7–16)	16 (12–18)	7 (0–10)	<0.001	0.729 (0.591–0.877)	0.001
Mitral leaflet tip—posterior wall	early-systolic	18 (16–21)	18 (15–21)	19 (17–21)	0.034	0.961 (0.831–1.111)	0.592
	late-systolic	15 (12–19)	13 (10–15)	18 (15–23)	<0.001	1.153 (1.002–1.327)	0.047

Data are frequency are mean (standard deviation) or median (quartiles).

Group comparisons performed using Mann–Whitney *U* test.

Multivariable logistic regression analyses with LVOTO as dependent variable and the respective spatial relationship parameter as independent variable. Covariates include sex, NYHA functional class, atrial fibrillation, device therapy, LVEF, degree of SAM, maximal septal thickness, apical displacement of anterolateral papillary muscle, and apical displacement of posteromedial papillary muscle.

AML, anterior mitral leaflet; IVS, interventricular septum; LVOTO, left ventricular outflow tract obstruction; OR, Odds ratio; PML, posterior mitral leaflet.

### Diagnostic accuracy analyses

#### LVOTO

Among spatial relationship parameters, late-systolic TIS showed the highest diagnostic accuracy for identification of LVOTO, with AUC of 0.91 (95% CI 0.87–0.96; *Table 3*). TIS was measurable in 137 patients (feasibility 96%). From AUC, optimized TIS thresholds of 14 and 9 mm were derived (Graphical Abstract). Among 63 patients with LVOTO and available TIS, 2 (5%) had TIS >14 mm, 15 (35%) had TIS between 10 and 14 mm, and 46 (92%) had TIS ≤9 mm. TIS ≤ 14 mm yielded 97% sensitivity and 57% specificity regarding LVOTO (diagnostic effectiveness 75%). TIS was >14 mm in 44 patients (31%) and ruled out LVOTO with a negative predictive value of 95% (*Table 4*). TIS was ≤9 mm in 50 patients (35%) and ruled in LVOTO with a positive predictive value of 92% (sensitivity 73%, specificity 95%, and diagnostic effectiveness 85%). Among 43 patients with TIS between 10 and 14 mm, 15 (35%) had LVOTO (*n* = 9 with previous SRT).

**Table 3:** Diagnostic accuracy of echocardiographic spatial relationship parameters identifying left ventricular outflow tract obstruction using ROC curve analyses

		LVOTO AUC (95% confidence interval)
**Lengths**		
Complete AML		0.63 (0.53–0.72)
Complete PML		0.68 (0.59–0.77)
Residual mitral leaflet	early-systolic	0.81 (0.74–0.88)
	late-systolic	0.81 (0.737–0.88)
**Distances**		
Coaptation point—ventricular septum#	early-systolic	0.64 (0.54–0.73)
	late-systolic	0.70 (0.61–0.79)
Coaptation point—posterior wall	early-systolic	0.59 (0.49–0.69)
	late-systolic	0.57 (0.47–0.66)
Mitral leaflet tip—ventricular septum (TIS)#	early-systolic	0.71 (0.62–0.80)
	late-systolic	0.91 (0.87–0.96)
Mitral leaflet tip—posterior wall	early-systolic	0.61 (0.51–0.70)
	late-systolic	0.82 (0.74–0.89)

# inverse association.

AML, anterior mitral leaflet; AUC, area under the ROC curve; LVOTO, left ventricular outflow tract obstruction; PML, posterior mitral leaflet; ROC, receiver-operating characteristics.

**Table 4:** Diagnostic accuracy of late-systolic mitral leaflet tip-to-septum distance (TIS) to identify left ventricular outflow tract obstruction

	No LVOTO *n* = 74	LVOTO *n* = 63	Sensitivity	Specificity	PPV	NPV
TIS >9 mm, *n* (%)	70 (95%)	17 (27%)	73.0%	94.6%	92.0%	80.5%
TIS ≤9 mm, *n* (%)	4 (5%)	46 (73%)				

TIS >14 mm, *n* (%)	42 (57%)	2 (3%)	96.8%	56.8%	65.6%	95.4%
TIS ≤14 mm, *n* (%)	32 (43%)	61 (97%)				

LVOTO, left ventricular outflow tract obstruction; NPV, negative predictive value; PPV, positive predictive value; TIS, late-systolic distance between mitral leaflet tip and interventricular septum.

#### Provocable LVOTO

The AUC of TIS to identify provocable LVOTO—omitting patients with resting LVOTO—was 0.87 (95% CI 0.79–0.93; Supplementary data online, *Figure S1*). Among 35 patients with provocable LVOTO and available TIS, 2 (6%) had TIS >14 mm, 14 (40%) had TIS between 10 and 14 mm, and 19 (54%) had TIS ≤9 mm. Diagnostic accuracy read-outs for TIS thresholds of 9 and 14 mm regarding provocable LVOTO are illustrated in *Table 5*.

**Table 5:** Diagnostic accuracy of late-systolic mitral leaflet tip-to-septum distance (TIS) to identify provocable left ventricular outflow tract obstruction

	No LVOTO *n* = 74	Provocable LVOTO *n* = 35	Sensitivity	Specificity	PPV	NPV
TIS >9 mm, *n* (%)	70 (95%)	16 (46%)	54.3%	94.6%	82.6%	81.4%
TIS ≤9 mm, *n* (%)	4 (5%)	19 (54%)				
TIS >14 mm, *n* (%)	42 (57%)	2 (6%)	94.3%	56.8%	50.8%	95.5%
TIS ≤14 mm, *n* (%)	32 (43%)	33 (95%)				

LVOTO, left ventricular outflow tract obstruction; NPV, negative predictive value; PPV, positive predictive value; SAM, systolic anterior motion of the mitral valve; TIS, late-systolic mitral leaflet tip-to-septum distance.

#### Subgroup analyses

The AUC of TIS for the presence of resting LVOTO—omitting 35 patients with provocable LVOTO—was 0.90 (95% CI 0.96–1.00). When confining analyses to those without previous SRT, TIS yielded an AUC of 0.95 (95% CI 0.91–0.99). The diagnostic accuracy of TIS was similar for both sexes: for women, AUC 0.92 (95% CI 0.85–0.99); for men, AUC 0.93 (95% CI 0.87–0.98). Indexing TIS for BSA minimally improved diagnostic accuracy: AUC 0.92 (95% CI 0.88–0.96). Indexing TIS for left ventricular stroke volume also slightly improved diagnostic accuracy: AUC 0.93 (95% CI 0.88–0.97). Representative measurements of TIS are shown in Supplementary data online, *Figure S2*.

#### Observer agreement

TIS was re-measured in 20 randomly selected patients by two cardiologists in a blinded fashion (NV, AB) to assess intra- and inter-observer agreement. TIS measurement required on average less than 1 min per patient. TIS showed very favourable intra- and inter-observer variability, with intra-class correlation coefficients of 0.96 (95% CI 0.82–0.99) and 0.95 (0.83–0.98), respectively. Expressing TIS in categories, intra-observer agreement was good regarding both thresholds: > 14 mm yielded κ=0.79 and agreement in 90%, while ≤9 mm yielded κ=0.70 and agreement in 85%. Further, there was good inter-observer agreement regarding the threshold >14 mm with κ=0.79 (agreement in 90%) and moderate inter-observer agreement regarding the threshold ≤9 mm with κ=0.50 (agreement in 75%).

## Discussion

In a well-characterized cohort of consecutive patients with HCM, we identified late-systolic TIS as an echocardiographic parameter that can be obtained under resting conditions and has high negative and positive predictive values for LVOTO in HCM. TIS has high feasibility when acquired in apical 3ch view and can be measured easily and with high reproducibility during routine echocardiography work-up.

### Relevance of LVOTO in HCM

In patients with HCM, LVOTO at rest increases morbidity and mortality.^2^ Both pharmacological therapies and SRT are effective in relieving LVOTO and associated symptoms.^5,7,21–23^ Of note, whereas provocable obstruction is as common as resting obstruction, it frequently remains undiagnosed. The execution of provocative manoeuvers for its correct diagnosis is time-consuming and requires experienced investigators.^3^ Although specific symptom assessment (worsening of symptoms after intake of food, even low amounts of alcohol, or drugs that decrease pre- or afterload) and physical examination (increase of systolic murmur after Valsalva manoeuver and in standing position) can be helpful, such derived diagnostic information is subjective by nature and depends on the experience of the individual physician.^24^ Yet, a diagnostic parameter excluding or indicating LVOTO and prompting provocative manoeuvers is lacking.

### Spatial relationship between mitral valve and ventricular septum

Systolic motion of the mitral valve leaflets towards the ventricular septum is the central mechanism underlying LVOTO in patients with HCM.^9^ Since the 1960s, the intricate interaction between ventricular septum and the mitral valve has been investigated. Authors combining mechanographic, haemodynamic, and angiographic methods and M-mode echocardiography described this interaction as SAM.^25^ Recent HCM guidelines, however, do not provide clear definitions how to quantify SAM.^9,14^ Also after SRT, residual LVOTO can be present.^8^ Spirito and colleagues showed that the diagnostic accuracy of semi-quantified SAM was low, as eight out of 10 patients with post-operatively provocable LVOTO had no SAM.^11^ Also in untreated HCM patients, SAM was not associated with presence of LVOTO in another study.^3^

In the context of septal hypertrophy and ventricular hypercontractility, both mitral leaflet elongation and apical papillary displacement are considered as most important factors permitting the mitral valve to protrude into the LVOT.^12^ Mitral valve alterations are well established as surgical treatment targets, but their quantitative assessment during the diagnostic work-up of HCM has not yet been implemented. Patients with HCM exhibit on average longer mitral leaflets than healthy persons, but the contributing role of total mitral leaflet length to LVOTO has been discussed controversially.^11,26^ Therefore, it was suggested to focus on the post-coaptational leaflet portion describing the residual leaflet portion distant to the mitral valve coaptation point.^12^ In line with this concept, patients admitted with acute takotsubo syndrome and LVOTO during the ballooning event exhibited significantly longer residual leaflets compared to those not developing LVOTO.^27^ In pivotal studies by Sherrid and colleagues, echocardiographic assessment of the post-coaptational portion relied on early-systolic measurements of residual leaflet length and distances between the mitral valve coaptation point and ventricular walls, all acquired in PLAX.^27^ Of note, the mobility of the tip of the residual leaflet makes it susceptible to systolic hydrodynamic forces. Therefore, we measured distances between mitral leaflet tip and ventricular walls, on top of measurements at the coaptation point, acquired both at early and late systole. Taking into account that SAM and LVOT gradients are often best visualized in 3ch view, we measured all parameters in 3ch view on top of PLAX. We identified late-systolic TIS measured at rest in 3ch view as an echocardiography-derived predictor of LVOTO with favourable diagnostic properties. This novel echocardiographic parameter advances the former concept and can be easily derived in clinical routine 2D echocardiography within less than min, either instantly or using post-processing software. Interestingly, a recent retrospective study applying machine learning algorithms in a large dataset of HCM patients with available cardiac magnetic resonance imaging reported concordant results.^28^ They found that among several anatomical parameters, distance between anterior mitral valve leaflet tip and basal septum best predicted presence of resting LVOTO. Our study further extends these findings involving also patients with provocable LVOTO and providing an easily applicable echocardiographic parameter.

Our study indicates that TIS between 10 and 14 mm represents an area of diagnostic uncertainty. Interestingly, all but one patient with LVOTO and TIS in this area had provocable LVOTO, and 60% had a previous SRT. Given our relatively small study population, subgroup analyses should be interpreted cautiously. Yet, our study suggests that TIS may perform substantially better in patients without previous SRT. The diagnostic accuracy of TIS in SRT-naïve patients with HCM and also the effect of SRT on the spatial relationship between mitral valve and ventricular septum should be addressed more specifically in prospective studies.

### Potential clinical implications

Our results may have important implications on diagnostic and therapeutic strategies. In HCM patients with a TIS >14 mm, the probability of an LVOTO appears so low that further provocation manoeuvers may be omitted and the search for alternative causes of symptoms should be initiated. Approximately 40% of patients with provocable LVOTO had TIS in the range of 9–14 mm. Therefore, in patients with TIS in this ‘grey zone’ and symptoms indicative of LVOTO, particular attention needs to be paid to provocation manoeuvers in order to uncover a potentially latent LVOTO. A TIS of ≤9 mm appears to raise the likelihood for the presence of a clinically relevant LVOTO close to 100%.

### Limitations

Due to the exploratory nature of the study design and analyses carried out *post hoc*, the performance of the identified TIS thresholds await verification in prospective larger studies. However, effect sizes were large, with fairly narrow confidence intervals and in line with a sound pathophysiologic concept. The mono-centric character may limit generalizability of our findings. Measurements were performed by a single experienced investigator, but TIS showed good inter-observer variability when re-assessed for validation purposes in a subset of patients.

## Conclusion

In our study, the novel echocardiographic parameter TIS showed high negative and positive predictive values regarding the presence LVOTO in HCM. Application of TIS in resting echocardiography protocols may help in tailoring the diagnostic work-up in HCM and identifying those patients who should or should not undergo echocardiography with extensive provocative manoeuvers. These exploratory results need to be confirmed in larger collectives and prospective investigations.

## Supplementary data

Supplementary data is available at *European Heart Journal - Cardiovascular Imaging* online.

## Supplementary Material

jead036_Supplementary_Data

## Data Availability

The data underlying this article will be shared on reasonable request to the corresponding author.
