# Supplementary figures and images for: Spatial relationship between mitral valve and ventricular septum assessed by resting echocardiography to diagnose left ventricular outflow tract obstruction in hypertrophic cardiomyopathy
Source: Eur Heart J Cardiovasc Imaging. 2023 Feb 26;24(6):710–8. doi: 10.1093/ehjci/jead036 (PMC10229295; doi:10.1093/ehjci/jead036)

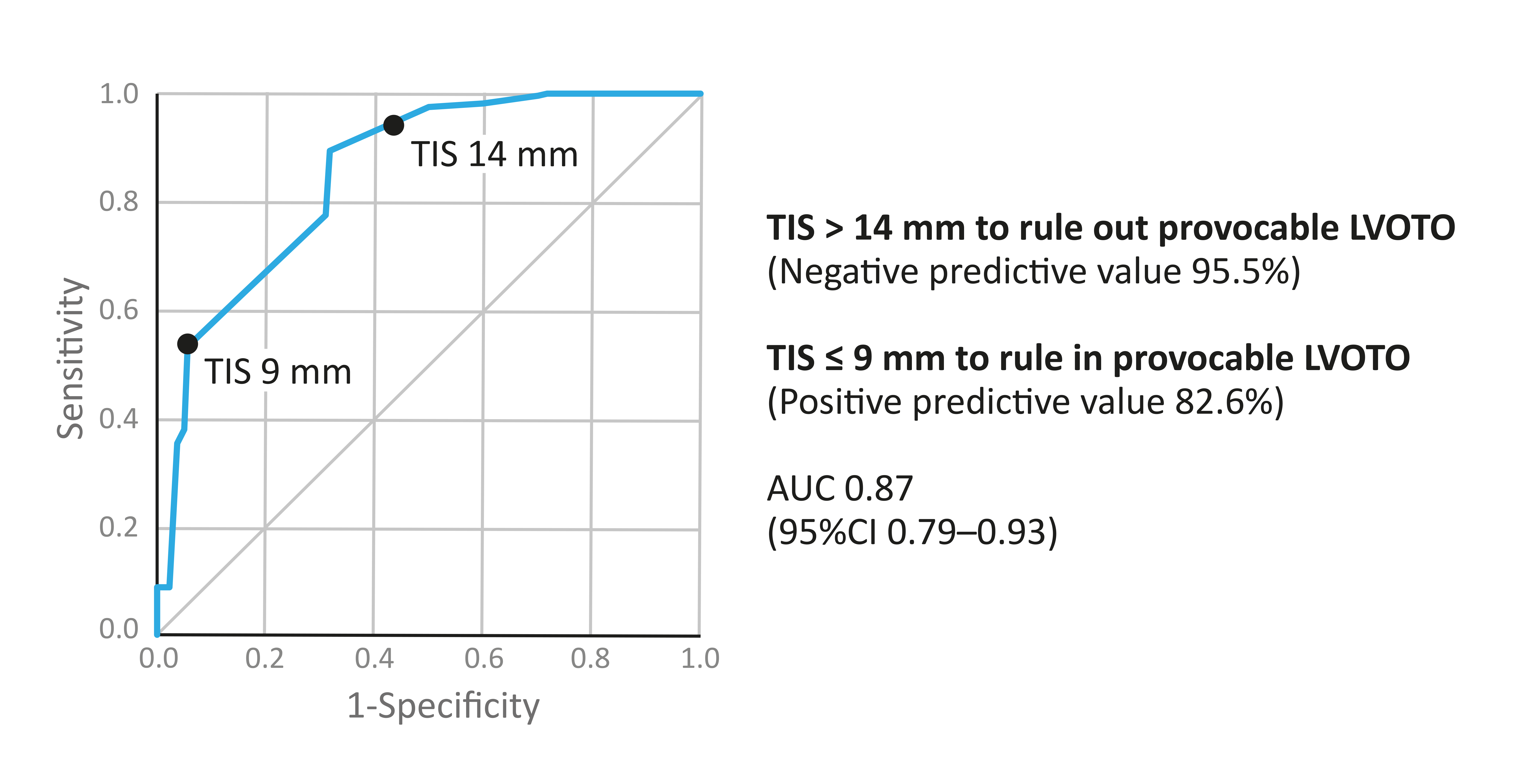

Supplement: jead036_Supplementary_Data [file jead036_supplementary_data.zip › Figure S1.tif]
